# Correction: Factors affecting the relapse of maxilla and soft tissues of nose, upper lip and velopharyngeal structures after maxillary advancement in cleft patients

**DOI:** 10.1371/journal.pone.0318829

**Published:** 2025-01-30

**Authors:** The PLOS ONE Staff

There are errors in the author affiliations. The correct affiliations are as follows:

Sirada Chaisiri^1^, Raweewan Arayasantiparb^2^, Kiatanant Boonsiriseth^1^

**1** Department of Oral and Maxillofacial Surgery, Faculty of Dentistry, Mahidol University, Bangkok, Thailand, **2** Department of Oral and Maxillofacial Radiology, Faculty of Dentistry, Mahidol University, Bangkok, Thailand.

The publisher apologizes for the error.

## References

[1] Chaisiri S, Arayasantiparb R, Boonsiriseth K (2023) Factors affecting the relapse of maxilla and soft tissues of nose, upper lip and velopharyngeal structures after maxillary advancement in cleft patients. PLOS ONE 18(11): e0294059. doi: 10.1371/journal.pone.0294059 37939044 PMC10631644

