# Supplementary material for: Factors affecting the relapse of maxilla and soft tissues of nose, upper lip and velopharyngeal structures after maxillary advancement in cleft patients
Source: PLoS One. 2023 Nov 8;18(11):e0294059. doi: 10.1371/journal.pone.0294059 (PMC10631644; doi:10.1371/journal.pone.0294059)
Supplement: Attachment — Submitted filename: Response to reviewers.pdf [file pone.0294059.s002.pdf]

Journal Submissions Rebuttal Letter

15 July 2023

Dear Editor

Thank you for inviting us to submit a revised draft of our manuscript entitled, "Factors affecting the relapse of maxilla and soft tissues of nose, upper lip and velopharyngeal structures after maxillary advancement in cleft patients" to PLOS ONE. We also appreciate the time and effort you and each of the reviewers have dedicated to providing insightful feedback. Thus, it is with great pleasure that we resubmit our article for further consideration. We have incorporated changes that reflect the detailed suggestions you have graciously provided. We also hope that our edits and the responses we provide below satisfactorily address all the issues and concerns you and the reviewers have noted.

Journal Requirements:

When submitting your revision, we need you to address these additional requirements.

1. Please ensure that your manuscript meets PLOS ONE's style requirements, including those for file naming. The PLOS ONE style templates can be found at https://journals.plos.org/plosone/s/file?id=wjVg/PLOSOne_formatting_sample_main_body.pdf and https://journals.plos.org/plosone/s/file?id=ba62/PLOSOne_formatting_sample_ title_ authors_affiliations.pdf.

As stated on the website that PLOS ONE waives all formatting requirements until your manuscript has received a provisional Editorial Accept decision. We intend to adjust and provide the files necessary as soon as the provisional Editorial Accept decision declares. It would be much appreciated if it's possible to submit the manuscript with the formatting according to the PLOS ONE guidelines thereafter.

2. Please provide additional details regarding participant consent. In the ethics statement in the Methods and online submission information, please ensure that you have specified (1) whether consent was informed and (2) what type you obtained (for instance, written or verbal, and if verbal, how it was documented and witnessed). If your study included minors, state whether you obtained consent from parents or guardians. If the need for consent was waived by the ethics committee, please include this information.

   If you are reporting a retrospective study of medical records or archived samples, please ensure that you have discussed whether all data were fully anonymized before you accessed them and/or whether the IRB or ethics committee waived the requirement for informed consent. If patients provided informed written consent to have data from their medical records used in research, please include this information.

This article is a retrospective study of medical records and radiographic images. The ethics committee waived the requirement for informed consent. We ensure that all data are fully anonymized.

Reviewer's Responses to Questions

Comments to the Author

1. Is the manuscript technically sound, and do the data support the conclusions?

The manuscript must describe a technically sound piece of scientific research with data that supports the conclusions. Experiments must have been conducted rigorously, with appropriate controls, replication, and sample sizes. The conclusions must be drawn appropriately based on the data presented.

Reviewer #1: Partly

Reviewer #2: Yes

Please refer to the table at the end of this letter for the responses to the reviewers.

---

2. Has the statistical analysis been performed appropriately and rigorously?

Reviewer #1: No

Reviewer #2: Yes

Please refer to the table at the end of this letter for the responses to the reviewers.

---

3. Have the authors made all data underlying the findings in their manuscript fully available?

The PLOS Data policy requires authors to make all data underlying the findings described in their manuscript fully available without restriction, with rare exception (please refer to the Data Availability Statement in the manuscript PDF file). The data should be provided as part of the manuscript or its supporting information, or deposited to a public repository. For example, in addition to summary statistics, the data points behind means, medians and

variance measures should be available. If there are restrictions on publicly sharing data—e.g. participant privacy or use of data from a third party—those must be specified.

Reviewer #1: No

Reviewer #2: Yes

We will attach the file containing all the data points as "supporting information" file when submitting the revised manuscript.

---

5. Review Comments to the Author

Please use the space provided to explain your answers to the questions above. You may also include additional comments for the author, including concerns about dual publication, research ethics, or publication ethics. (Please upload your review as an attachment if it exceeds 20,000 characters)

For convenience purpose, we arranged review comments to the author, author responses, and corrections in the revised manuscript sectioned into topics listed in the table at the end of this letter.

CONCLUDING REMARKS: We thank you again for giving us the opportunity to strengthen our manuscript with your valuable comments and queries. We have worked hard to incorporate your feedback and hope that these revisions persuade you to accept our submission.

Sincerely.
Dr. Kiatanant Boonsiriseth

Corresponding author:
Kiatanant Boonsiriseth
Email: kiatanant.boo@mahidol.ac.th
Address: Department of Oral and Maxillofacial surgery, Faculty of Dentistry, Mahidol University. No. 6, Yothi Road, Ratchathewi District, Bangkok, Thailand, 10400
Telephone: (662) 200-7845-6

Topic	Reviewer comments	Author responses	Correction in the revised manuscripts
Reviewer #1: The primary aim of this study was to identify the factors associated with hard-and soft-tissue relapse following maxillary advancement in cleft patients. While the study's objectives are intriguing, there are several concerns that need to be addressed to enhance the quality of the research. In particular, it is important for the authors to consider potential confounding factors and choose appropriate statistical methods. Here are some specific recommendations:			
1	The authors should provide details about the selection method used for distinguishing between distraction osteogenesis (DO) or conventional orthognathic surgery (CO), as well as isolated or bimaxillary surgery. It is crucial to determine if the authors used a uniform subject group for DO and CO procedures. Additionally, the authors should carefully consider potential confounding factors that may influence the outcomes.	Thank you for your suggestion. Primarily, the decision whether the patient would undergo CO or DO, isolated maxillary or bimaxillary surgery was based on the severity of the skeletal deformity and the restriction of the soft tissue scar. Other characteristics such as the available posterior airway, and the relative mandibular deformity had effects on the surgical treatment plan individually. In our opinion, the indications/selection methods cannot be defined definitely or numerically, therefore, the restrictive criteria to justify subject grouping were impractical. However, we sincerely acknowledge the inadequate information on the selection method and attempt to improve by adding the overall consideration for selecting CO or DO, and isolated or bimaxillary surgery.<br>Our study primarily aims to evaluate each factor separately. We are aware that the potential confounding factors may interfere with the outcomes of the individual factor. Unfortunately, the combined effects of factors are beyond the purpose of this study. Future studies of fewer focused factors with a tight control of potential confounding factors are required.	**Page 4 and 5**, under “Surgical technique” section

Topic	Reviewer comments	Author responses	Correction in the revised manuscripts
2	In the Method section, the authors refer to "Factor Analysis" in the title. However, factor analysis is a statistical technique used to reduce a large number of variables into a smaller set of factors, and it may not be appropriate for this study. If the authors performed correlation analysis instead, they should clearly state it.	As stated in statistical analysis paragraph (page 8), this study either compare between two groups with different factors or evaluate correlations between two factors if the factor was a continuous data. We change "Factor Analysis" to "Data analysis" in this section to represent overall method for evaluating each factor.	Page 7 change "Factor Analysis" to "Data analysis"
3	Furthermore, when describing the correlations between variables, it would be helpful to classify the strength of the correlations as strong, moderate, or weak based on the corresponding r values.	We add the strength of the correlations based on r values as suggested.	Added in result section (page 15,16), and discussion section (page 17)
4	Since the authors are examining multiple factors, it is advisable to employ multivariate analysis to identify the variables that significantly influence the relapse rate.	We absolutely agree. Unfortunately, the number of the patients recruited in this study was simply inadequate for multivariate analysis. We added recommendation for future multivariate study.	Page 17
5	The use of ICC (Intraclass Correlation Coefficient) alone is insufficient to demonstrate reliability. It is recommended to present additional measures such as absolute differences, standard deviation, and coefficient of variation to provide a comprehensive understanding of the reliability of the study findings.	We appreciate the recommendation and have adjusted the ICC report by including intra-class correlation coefficient (ICC) and absolute difference (mean±SD) as suggested.	Page 10, Table 3 Intra-class correlation coefficient (ICC) and absolute difference (mean±SD) between the two measurements of the examiner

<table>
<tr><th>Topic</th><th>Reviewer comments</th><th>Author responses</th><th>Correction in the revised manuscripts</th></tr>
<tr><td>6</td><td>The authors should explain the methodology used to determine the cut-off value for classifying significant relapse or non-significant relapse.</td><td>As stated in page 7 under the paragraph “Association between skeletal relapse and soft tissue relapse”. Patients who had significant skeletal relapse had ≥2 mm. of horizontal relapse. The criteria is from a study by Proffit et al.(32) where changes of less than 2 mm were considered within the range of method error and clinically insignificant. Changes of ≥2 mm were outside the range of method error and potentially clinically significant.</td><td>-</td></tr>
<tr><td>7</td><td>In Table 5, the number of patients appears to be relatively small. It is important to consider the sample size and potential implications for the statistical power of the study.</td><td>We agree that the sample size was fairly small despite including all the patients who met the criteria during the ten-year period at our faculty. Nevertheless, the sample size had met the minimum of the sample size determined based on the results of the comparable previous study. (shown in fig. below)
Sample size determination
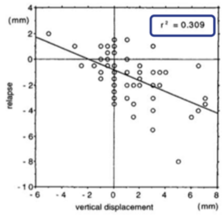

FIGURE 2 Significant relation ($p < .05$) between vertical surgical displacement and relapse after 1 year.
Factors Related to Relapse After Le Fort I Maxillary Advancement Osteotomy in Patients With Cleft Lip and Palate
Akiyoshi Hirano, M.D.
Hiroyuki Suzuki, D.D.S.
$C(r) = \frac{1}{2} ln[\frac{1+r}{1-r}]$
$n = [\frac{Z_\alpha + Z_\beta}{C(r)}]^2 + 3$
$n = [\frac{(1.96+0.842)}{0.629}]^2 + 3 = 22.97 \sim 23$</td><td>-</td></tr>
</table>

Topic	Reviewer comments	Author responses	Correction in the revised manuscripts
Reviewer #2: The manuscript explains several factors affecting skeletal and soft tissue relapse in maxillary advancement surgery in cleft patients.<br>The data presented has been able to justify the findings. Nevertheless, the sample size is not robust enough for the findings to be universally applicable.<br>Additionally, the data would have been more accurate if a 3-dimensional modality such as CBCT would have been used for measurement.		Thank you for your valuable comments. We agree that the sample size was small. Please kindly refer to question 7 in this table for further information.<br>We are aware that the small sample size is our limitation. However, the study has shown some notable results, for example, the correlation between skeletal relapse rate and skeletal advancement, the significant amount of skeletal relapse did not affect the amount of soft tissue relapses, the correlations between the nose and upper lip soft tissue and the changes of UIT position. These results raise awareness of the hard and soft tissue responses which may or may not coincide, and certainly, the factors that may affected them.<br>The CBCT during the early period of this study was not as widely accessible as it is now. With the more accessible CBCT for cleft patients, we surely agree that CBCT data would provide a more accurate and in-depth evaluation in future studies.	
