# Supplementary material for: Factors affecting the relapse of maxilla and soft tissues of nose, upper lip and velopharyngeal structures after maxillary advancement in cleft patients
Source: PLoS One. 2023 Nov 8;18(11):e0294059. doi: 10.1371/journal.pone.0294059 (PMC10631644; doi:10.1371/journal.pone.0294059)
Supplement: Attachment — Submitted filename: Response to reviewers.docx [file pone.0294059.s003.docx]

**Journal Submissions Rebuttal Letter**

6 September 2023

Dear Editor

Thank you again for the time and effort you and each of the reviewers have dedicated to providing insightful feedback for our manuscript entitled, "Factors affecting the relapse of maxilla and soft tissues of nose, upper lip and velopharyngeal structures after maxillary advancement in cleft patients". We have incorporated changes that reflect the detailed suggestions you have graciously provided. We also hope that our edits and the responses we provide below satisfactorily address all the issues and concerns you and the reviewers have noted.

**Journal Requirements:**

Please review your reference list to ensure that it is complete and correct. If you have cited papers that have been retracted, please include the rationale for doing so in the manuscript text, or remove these references and replace them with relevant current references. Any changes to the reference list should be mentioned in the rebuttal letter that accompanies your revised manuscript. If you need to cite a retracted article, indicate the article’s retracted status in the References list and also include a citation and full reference for the retraction notice.

We have reviewed our reference list and there were no retracted papers cited.

**Reviewer's Responses to Questions**

**Comments to the Author**

**Reviewer #1:**

1. The selection of the samples is paramount for this study since the DO group could have severe status according to the reviewer's comments, which can be a bias for the results. At least, the authors should incorporate another statistical analysis to cancel any potential bias.

We thank you again for giving us the opportunity to strengthen our manuscript with your valuable comments and queries. We tried our best to reduce the potential bias from the potential confounding factor “the amount of skeletal advancement”. The ANCOVA analysis was used instead of t-test/Mann Whitney U test to control/reduce the effects of the amount of advancement when comparing skeletal relapse. Not only between CO and DO group, but also other factors were revised. Unfortunately, after ANCOVA analysis was used, no significant difference of skeletal relapse rates between two groups of patients with different factors, including between patients underwent maxillary advancement by CO and DO.

According to the revision above, we updated table 5 in our manuscript along with minor adjustments in statistical analysis, result, and discussion parts. Please refer to the revision details mentioned below.

1. (Page 9) Statistical analysis part was revised by adding the ANCOVA analysis along with minor context refinements.
2. (Page 12) In the result part, minor refinement at the introduction sentence before comparing skeletal relapse between groups with ANCOVA analysis.
3. (Page 13) Table 5 was updated as listed below

- As the amount of advancement has been included in the ANCOVA analysis and for the simplicity of the table. the amount of skeletal advancement column was removed. The title of the table has been changed from “Compare horizontal skeletal relapse rates and amount of skeletal advancement between two groups of patients with different factors” to “Compare horizontal skeletal relapse rates between two groups of patients with different factors”
- Revised P-value after the ANCOVA tests was used.
- Notations at the end of the table were revised.

4. (Page 19) In the discussion, part “Factors affecting skeletal relapse after cleft orthognathic surgery”, paragraph 3, we focused on the factor CO vs DO. Minor contexts were refined by adding the mean amount of skeletal advancement between CO vs DO and isolated maxillary vs bimaxillary surgery)
2. Furthermore, the rationale behind the chosen number of patients must be thoroughly described in the manuscript, rather than simply displaying the figures in the comments.

Thank you very much for the kind reminder. We have made revisions accordingly. The rationale behind the chosen number of patients has been added in the revised manuscript (Page 4-5).

3. From Section Reviewer comments to author, Topic 2.

Is the manuscript technically sound, and do the data support the conclusions?

The manuscript must describe a technically sound piece of scientific research with data that supports the conclusions. Experiments must have been conducted rigorously, with appropriate controls, replication, and sample sizes. The conclusions must be drawn appropriately based on the data presented.

Reviewer #1: Partly

Other than the revision made above, we refined the conclusion between CO group vs DO group to accurately reflect the results. (Page 25)

**Reviewer #2**: The study attempted to look for factors associated with skeletal relapse after maxillary advancement in cleft patients along with the correlation with soft tissue relapses. The study seems to be properly planned and executed and would serve as a valuable addition to the literature pertaining to cleft and craniofacial surgery.

Thank you kindly. We really appreciate you taking the time to share your comments with us.

Sincerely.

Dr. Kiatanant Boonsiriseth

Corresponding author:

Kiatanant Boonsiriseth

Email: kiatanant.boo@mahidol.ac.th

Address: Department of Oral and Maxillofacial surgery, Faculty of Dentistry, Mahidol University. No. 6, Yothi Road, Ratchathewi District, Bangkok, Thailand, 10400

Telephone: (662) 200-7845-6
